# BUILDING BRIDGES POST-LICENSURE: DEVELOPING GEROPSYCHOLOGY CONSULTATION SERVICES

**DOI:** 10.1093/geroni/igac059.300

**Published:** 2022-12-20

**Authors:** Erin Emery-Tiburcio, Erin Kube, Cecilia Poon, Ann Steffen

**Affiliations:** Rush University Medical College, Chicago, Illinois, United States; VA Salt Lake City Healthcare System/ VISN 19 Clinical Resource Hub, Salt Lake City, Utah, United States; Nebraska Medicine, Omaha, Nebraska, United States; University of Missouri-St. Louis, St. Louis, Missouri, United States

## Abstract

More than one third of psychologists report frequently treating older adults, while only 1% consider themselves geropsychologists. Continuing education opportunities in geropsychology have historically been scant, with even fewer opportunities for expert case consultation in work with older adults. To identify interest in geropsychology consultation, the Building Bridges Post-Licensure (BBPL) group conducted an informal survey of licensed psychologists via listservs. Results among 80 respondents indicated strong interest in group consultation regarding foundational knowledge, assessment, and intervention. This presentation will describe the BBPL group's efforts to examine effective models of consultation, develop a consultation model for geropsychology, and partner with both professional and community-based organizations to develop the infrastructure for geropsychology consultation.

